# Prenatal Diagnosis of Microdeletions or Microduplications in the Proximal, Central, and Distal Regions of Chromosome 22q11.2: Ultrasound Findings and Pregnancy Outcome

**DOI:** 10.3389/fgene.2019.00813

**Published:** 2019-08-30

**Authors:** Shuyuan Li, Xu Han, Mujin Ye, Songchang Chen, Yinghua Shen, Jianmei Niu, Yanlin Wang, Chenming Xu

**Affiliations:** ^1^International Peace Maternity and Child Health Hospital, School of Medicine, Shanghai Jiao Tong University, Shanghai, China; ^2^Shanghai Key Laboratory of Embryo Original Diseases, Shanghai, China; ^3^Institute of Embryo-Fetal Original Adult Disease, Shanghai Jiao Tong University School of Medicine, Shanghai, China

**Keywords:** 22q11.2, microdeletions, microduplications, prenatal diagnosis, genetic counseling

## Abstract

Several recurrent microdeletions and microduplications in the proximal, central, and distal regions of chromosomal 22q11.2 have been identified. However, due to a limited number of patients reported in the literature, highly variable clinical phenotypes, and incomplete penetrance, the pathogenicity of some microdeletions/microduplications in 22q11.2 central and distal regions is unclear. Hence, the genetic counseling and subsequent pregnancy decision are extremely challenging, especially when they are found in structurally normal fetuses. Here, we reported 27 consecutive cases diagnosed prenatally with 22q11.2 microdeletions or microduplications by chromosomal microarray analysis in our center. The prenatal ultrasound features, inheritance of the microdeletions/microduplications, and their effects on the pregnancy outcome were studied. We found that fetuses with 22q11.2 microdeletions were more likely to present with structure defects in the ultrasound, as compared with fetuses with 22q11.2 microduplications. Both the prenatal ultrasound findings and the inheritance of the microdeletions/microduplications affected the parent’s decision of pregnancy. Those with structure defects in prenatal ultrasound or occurred *de novo* often resulted in termination of the pregnancy, whereas those with normal ultrasound and inherited from healthy parent were likely to continue the pregnancy and led to normal birth. Our study emphasized that proximal, central, and distal 22q11.2 deletions or duplications were different from each other, although some common features were shared among them. More studies are warranted to demonstrate the underlying mechanisms of different clinical features of these recurrent copy-number variations, thereby to provide more information for genetic counseling of 22q11.2 microdeletions and microduplications when they are detected prenatally.

## Introduction

**Figure 1:**
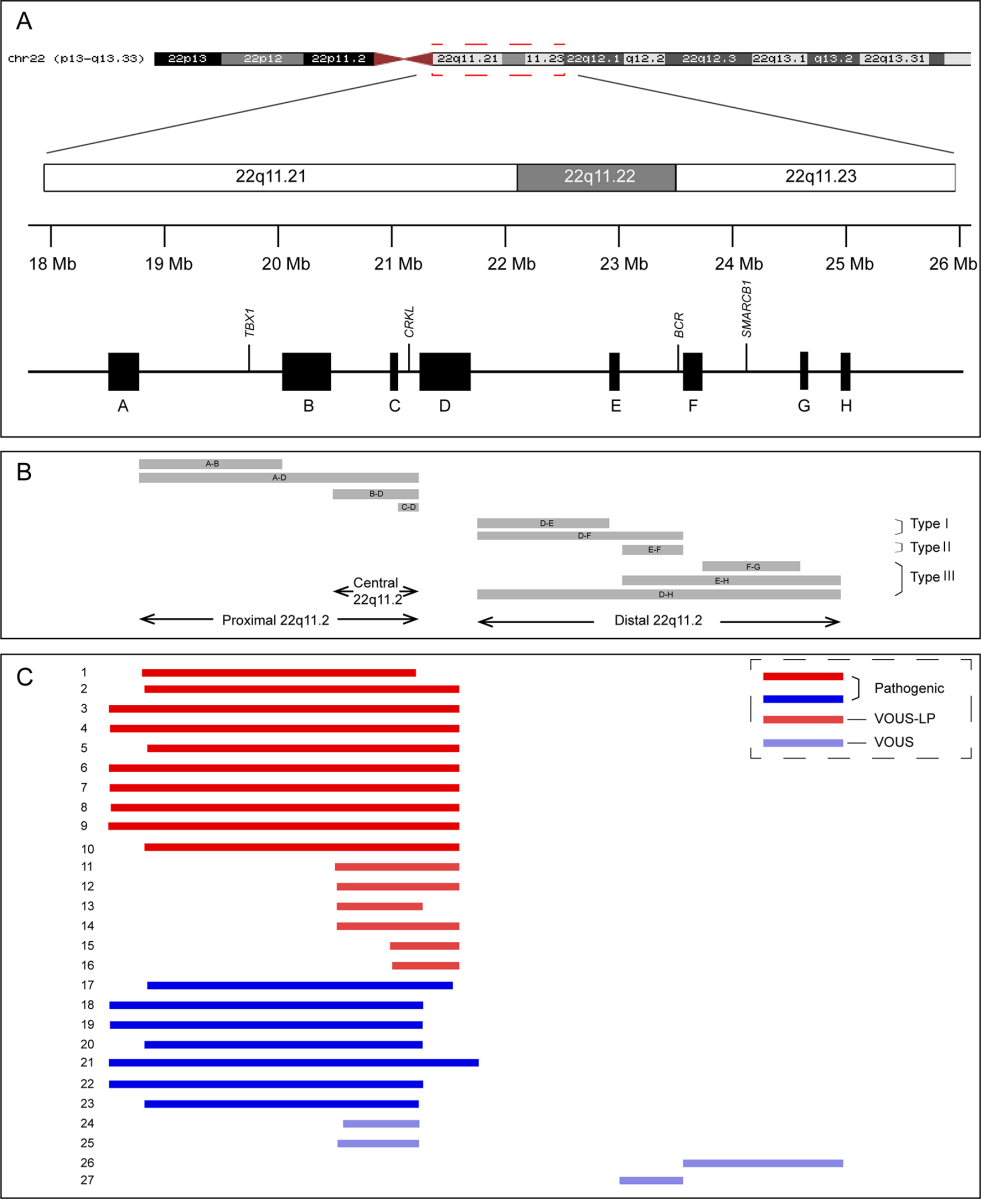
Schematic representation of chromosome 22q11.2 region **(A)**, the recurrent copy number variations reported in this region **(B)**, and the 27 cases included in this study **(C)**. VOUS, variant of unknown significance; LP, likely pathogenic.

Low copy repeats (LCRs), also known as segmental duplications, are highly homologous sequence (greater than 95% sequence identity) and comprise approximately 4–5% of the human genome (Bailey et al., 2001). Misalignment of LCRs during meiosis through the well-established mechanism of nonallelic homologous recombination (NAHR) can lead to recurrent copy-number variations (CNVs), including microdeletions and microduplication. When dosage-sensitive gene(s) involved, the microdeletions or microduplications may result in abnormal phenotypes (Dittwald et al., 2013). Eight LCRs, naming LCR22A-H, have been identified in chromosome 22q11.2, one region showing high frequency of genomic rearrangement (Shaikh et al., 2007). Several recurrent microdeletions and microduplications in chromosome 22q11.2 have been identified, including chromosome 22q11.2 deletion syndrome [also known as DiGeorge syndrome (#188400) or velocardiofacial syndrome (#192430), hereafter “22q11.2DS”], chromosome 22q11.2 deletion syndrome, distal (#611867), chromosome 22q11.2 duplication syndrome (#608363), and some others not recorded in the Online Mendelian Inheritance in Man database (22q11.2 central deletion or duplication, 22q11.2 distal duplication, etc.) (**Table 1** and **Figure 1**) (Burnside, 2015).

**Table 1:** Reported microdeletions or microduplications in chromosomal 22q11.2.

	LCR	Chromosome physical location (hg19)	OMIM syndrome	Haploinsufficiency/Triplosensitivity score ^a^	Classification of pathogenicity
**22q 11.2 microdeletion**					
Proximal	A-B/D	18,912,231-20,287,208/21,465,672	Chromosome 22q11.2 deletion syndrome: DGS (#188400) or VCFS (# 192430)	3	Pathogenic
Central	B/C-D	20,731,986-21,465,672	/	2	VOUS-LP
Distal Type I	D-E/F	21,917,117-23,649,111	Chromosome 22q11.2 deletion syndrome, distal (#611867)	3	Pathogenic
Type II	E-F	23,119,414-23,649,111	/	2	VOUS-LP
Type III	F-G	23,831,202-24,632,821	/	NA	VOUS
	E-H	23,119,414-24,994,433	/	NA	VOUS
	D-H	21,917,117-24,994,433	Chromosome 22q11.2 deletion syndrome, distal (#611867)	NA	Pathogenic
**22q11.2 microduplication**					
Proximal	A-B/D	18,912,231-20,287,208/21,465,672	Chromosome 22q11.2 duplication syndrome (#608363)	3	Pathogenic
Central	B/C-D	20,731,986-21,465,672	/	1	VOUS
Distal Type I	D-E/F	21,917,117-23,649,111	/	3	Pathogenic
Type II	E-F	23,119,414-23,649,111	/	1	VOUS
Type III	F-G	23,831,202-24,632,821	/	NA	VOUS
	E-H	23,119,414-24,994,433	/	NA	VOUS
	D-H	21,917,117-24,994,433	/	NA	Pathogenic

a Haploinsufficiency score (for deletions) and triplosensitivity score (for duplications) of the region curated in the ClinGen Dosage Sensitivity Map (https://www.ncbi.nlm.nih.gov/projects/dbvar/clingen). LCR, low copy repeats; DGS, DiGeorge syndrome; VCFS, velocardiofacial syndrome; NA, not available; VOUS, variant of unknown significance; LP, likely pathogenic; OMIM, Online Mendelian Inheritance in Man (www.omim.org).

The 22q11.2DS is the most common recurrent microdeletions in humans with a frequency estimated at 1:4,000 to 1:8,000 live births (McDonald-McGinn et al., 2015). The clinical features of patients with 22q11.2DS are variable and include cardiac defects, palatal abnormalities, characteristic facial features, learning difficulties, and immune deficiencies (Bassett et al., 2011). Approximately 85–90% of patients with 22q11.2DS result from a 3-Mb deletion extending from LCR22A to LCR22D, while 10–15% of the patients have a smaller “nested” ∼1.5-Mb deletions involving LCR22A to LCR22B (McDonald-McGinn et al., 2015). The *TBX1* (*602054) gene, located between the LCR22A and LCR22B, is the main candidate gene responsible for most of the features of 22q11.2DS, and the phenotypes of patients with LCR22A-B and LCR22A-D deletions are clinically indistinguishable (Carlson et al., 1997). Microduplications of the same region as 22q11.2DS have also been reported and are defined as chromosome 22q11.2 duplication syndrome (#608363) (Ensenauer et al., 2003; Hassed et al., 2004). The phenotypes of individuals with chromosome 22q11.2 duplication syndrome are highly variable, which range from apparently normal to severe malformations with developmental delay (Hassed et al., 2004; Wentzel et al., 2008). Some rare atypical deletions/duplications of shorter size, mainly involved LCR22B-D or LCR22C-D and not encompassed the *TBX1* gene, have been reported and now are proposed as “central” 22q11.2 deletions/duplications (Burnside, 2015; Rump et al., 2014). The “distal” 22q11.2 deletions/duplications, mediated by NAHR of the five distal LCR22s, LCR22D-H, have also been demonstrated (Ben-Shachar et al., 2008). Although the patients with central or distal 22q11.2 deletions/duplications share some characteristic features with 22q11.2DS, they have unique clinical characterizes with high phenotypic variability (Burnside, 2015; Rump et al., 2014).

Chromosomal microarray analysis (CMA) is a high-resolution technology capable of detecting aneuploidy, as well as microduplications and microdeletions, throughout the genome. The use of CMA in prenatal diagnosis has been recommended by the American College of Obstetricians and Gynecologist in 2013 (American College of Obstetricians and Gynecologists Commitee on Genetics, 2013). With the wide use of CMA in prenatal diagnosis, more and more fetuses with 22q11.2 microdeletions or microduplications with variable sizes have been identified. It has been reported that the prevalence of 22q11.2DS in fetuses with congenital heart defects is as high as 7% (Mademont-Soler et al., 2013). However, due to a limited number of patients reported in the literature, highly variable clinical phenotypes, and incomplete penetrance, the pathogenicity of microdeletions/microduplications in 22q11.2 central and distal regions (types II and III) is unclear (variants of unknown significance) (**Figure 1** and **Table 1**). Hence, the genetic counseling and subsequent pregnancy decision are extremely difficult, especially when they are found in structurally normal fetuses.

Here, we reported 27 consecutive cases diagnosed prenatally with 22q11.2 microdeletions or microduplications by CMA from December 2015 to September 2018 in our center. The prenatal ultrasound features, inheritance of the CNVs, and their effects on the pregnancy outcome were studied. Our study will provide more information for genetic counseling of 22q11.2 microdeletions and microduplications in prenatal diagnosis.

## Methods

### Study Population

This study was conducted in the Reproductive Genetic Center of International Peace Maternal and Child Health Hospital (IPMCH) of Shanghai Jiao Tong University School of Medicine. From December 2015 to September 2018, 5,464 pregnant women received an invasive prenatal diagnostic test for CMA analysis in our center. Among them, 16 fetuses of 22q11.2 microdeletion (0.29%) and 11 fetuses of 22q11.2 microduplication (0.20%) were detected by CMA. According to the gestational age (range: 12–28 weeks, median: 22 weeks), fetal samples were obtained using chorionic villus sampling (n = 1), amniocentesis (n = 21), or cord blood sampling (n = 5). All cases diagnosed with 22q11.2 microdeletions or microduplications were further consulted regarding the prognosis and additionally followed up for the clinical outcome. Written informed consent was obtained from the parents in accordance with the Declaration of Helsinki, and the study was approved by the Ethics Committee of the IPMCH (number of Institutional Review Board approval: GJEC-A-2015-11-1).

### Chromosomal Microarray Analysis and Quantitative Real-Time PCR

Genomic DNA was isolated according to standard procedures (Li et al., 2019). CMA was performed using Agilent 4X180K SurePrint Prenatal Research Array (Agilent Technologies, Santa Clara, CA,USA) from December 2015 to August 2016 (n = 2) and using Affymetrix CytoScan 750K Array (Affymetrix, Inc., Santa Clara, CA, USA) from September 2016 to September 2018 (n = 25). CNVs were determined using Agilent CytoGenomics (Agilent Technologies, Santa Clara, CA, USA) or Affymetrix Chromosome Analysis Suite software 3.2 (Affymetrix, Inc., Santa Clara, CA, USA), depending on the platform that was used. All results were evaluated using the University of California Santa Cruz human Genome Browser release of February 2009 (GRCh37/hg19). When parental blood samples were available, the inheritance of the detected microdeletions or microduplications of 22q11.2 was determined using CytoScan^®^ 750K Array or real-time quantitative PCR (qPCR). The qPCR was performed in a LightCycler 480 II (Roche Applied Science, Mannheim, Germany) qPCR machine according to the manufacturer’s instructions. The *HBB* gene was used as housekeeping gene, and the qPCR primers were shown in **Table S1**. Each sample was analyzed in triplicate. The temperature condition for qPCR was 95°C for 5 min; followed by 40 cycles consisting of 95°C for 15 s and 60°C for 1 min.

### Cytogenetic Analysis

A G-banding karyotype analysis was also performed in all 27 fetal samples. Twenty metaphase cells were checked for numerical abnormalities of chromosomes, and five metaphase cells were carefully examined to detect structural chromosomal abnormalities.

### Statistical Analysis

Categorical variables were summarized as number (percent) and were compared using chi-square test or Fisher’s exact test. All analyses were performed using SAS statistical software (release 9.3, SAS Institute Inc., Cary, NC, USA), and *P* value < 0.05 was considered to be statistically significant.

## Results

### Chromosomal Microarray Analysis and Karyotyping Result

Among the 16 cases diagnosed with 22q11.2 microdeletions, 10 of them were diagnosed with 22q11.2DS (proximal 22q11.2 region, LCR22-A to -D deletion, patient 1-10), and 6 others had deletions in the central 22q11.2 region 4 of LCR22-B to -D deletions (patients 11–14) and 2 of LCR22-C to -D deletions (patients 15–16). With respect to the 11 cases of 22q11.2 microduplications, 7 cases were detected with duplications in the proximal 22q11.2 region (LCR22-A to -D duplications, patient 17-23), 2 cases had duplications in the central 22q11.2 region (LCR22-B to -D duplications, patient 24-25), and 2 cases had duplications in the distal 22q11.2 region 1 of LCR22-F to -G duplications (patient 26), 1 of LCR22-E to -F duplications (patient 27). All the prenatal samples revealed normal karyotypes (**Table 2**, **Table S2**, and **Figure 1**).

**Table 2:** Characteristics of cases with prenatally diagnosed microduplications or microdeletions of 22q11.2 in this study.

	LCR	N	abnormal US^a^, n (%)	*De novo* ^b^, n (%)	TOP, n (%)
**22q11.2 microdeletion**					
Proximal	A-B/D	10	10 (100)	8 (80)	10 (100)
Central	B/C-D	6	4 (66.7)	0 (0)*^b^*	4 (66.7)
**22q11.2 microduplication**					
Proximal	A-B/D	7	1 (14.3)	4 (57.1)*^b^*	4 (57.1)
Central	B/C-D	2	0 (0)	0 (0)	0 (0)
Distal type II+ III	E-F/F-G	2	0 (0)	0 (0)*^b^*	0 (0)

a defined as structure defects.

b the inheritance was unknown in 6 fetuses, including 3 fetuses with central deletions, 2 fetuses with proximal duplications, and 1 fetus with distal duplications. US, ultrasound; TOP, termination of pregnancy.

### Prenatal Ultrasound Findings

Compared with fetuses with 22q11.2 microduplications, fetuses with 22q11.2 microdeletions were more likely to present with structure defects in the ultrasound. As shown in **Table 2** and **Table S2**, all the 10 fetuses (100%) with 22q11.2DS had abnormal prenatal ultrasound findings, including congenital heart defect (n = 7), multiple congenital abnormalities (n = 2), and congenital renal agenesis (n = 1). Abnormal ultrasound findings were also observed in four of the six fetuses (66.7%) with central 22q11.2 deletion, including congenital heart defect (n = 2), congenital anomaly of nervous system (n = 1), and multiple congenital abnormalities (n = 1). In contrast, only one of the seven fetuses (14.3%) with proximal 22q11.2 microduplications was detected with defects by prenatal ultrasound scan. Moreover, three of the seven fetuses with proximal 22q11.2 microduplications showed increased nuchal translucency in the first trimester ultrasound (≥3.0 mm), and one of the two fetuses with distal 22q11.2 microduplications presented with echogenic bowel in the second trimester ultrasound. All the other cases were presented with normal ultrasound.

### Inheritance

The parental samples were available in 21 cases, including 13 cases with microdeletions and 8 cases with microduplications (**Table 2** ****and **Table S2**). Eight of 10 cases (80%) with proximal deletions and 4 of 7 cases (57.1%) with proximal duplications occurred *de novo*. While, all other cases, including two fetuses with proximal deletions (one paternal and one maternal), one fetus with proximal duplications (paternal), three fetuses with central deletions (maternal), two fetuses with central duplications (one paternal and one maternal), and one fetus with proximal duplications (paternal) were inherited from parents. All the parents of the fetuses with inherited 22q11.2 microdeletions/microduplications were phenotypic normal, except the father of fetus 10 who presented with congenital atrial septal defect and mild developmental delay. In the remaining six cases, the inheritance could not be established, as the parents were not available for (or did not agree to) testing.

### Pregnancy Outcome

Eighteen cases resulted in the induced termination of pregnancy (TOP), including 10 cases (100%) with proximal 22q11.2 deletions, 4 of 6 cases (66.7%) with 22q11.2 central deletions, and 4 of 7 cases (57.1%) with proximal 22q11.2 duplications (**Table 2**). The effect of abnormal ultrasound findings and the inheritance of the deletions or duplications on the rate of TOP were examined. As shown in **Table 3**, the rate of TOP in the cases with abnormal ultrasound was significantly higher than that in the cases presented with normal ultrasound (100 vs 25%, *P* = 4 × 10^-5^). Before the decision was made, the inheritance of the CNVs was available in only nine fetuses (**Table S2**). In these fetuses, the rate of TOP was associated with the inheritance of the deletions or duplications (100% in the *de novo* group vs 25% in the inherited group, *P* = 0.02) (**Table 3**).

**Table 3:** Effect of ultrasound features, inheritance of the microdeletions, or microduplications on pregnancy outcome.

		N	TOP, n (%)	P*
Ultrasound	Abnormal^$^	15	15 (100)	4×10^-5^
	Normal	12	3 (25)	
Inheritance	*De novo*	4	4 (100)	0.02
	Inherited	5	1 (25)	

*chi-square test or Fisher’s exact test.

^$^defined as structure defect.

TOP, termination of pregnancy.

To clarify the independent effect of the inheritance on the pregnancy outcome, the TOP rate was further analyzed in fetuses with normal ultrasound. We found that all the four fetuses with normal ultrasound and inherited from healthy parent were born normally, whereas all the two fetuses with normal ultrasound and occurred *de novo* resulted in induced TOP (**Table S2**).

## Discussions

The application of CMA in prenatal diagnosis has greatly improved the detection rate of recurrent microdeletions and microduplications, which are common causes of congenital anomalies and neuropsychiatric disorders (Cooper et al., 2011; Grati et al., 2015). However, it is also accompanied by the detection of some CNVs with uncertain clinical significance, which may lead to great challenges in genetic counseling and parental anxiety. In this study, we reported on 27 new prenatally diagnosed cases of microdeletions or microduplications in the proximal (10 deletions and 7 duplications), central (6 deletions and 2 duplications), and distal (2 duplications) of chromosome 22q11.2, with particular attention being paid to the prenatal ultrasound findings and the pregnancy outcome of these fetuses.

Numerous studies about 22q11.2DS have been reported. Although the phenotypes of patients with 22q11.2DS are variable, the penetrance is nearly complete (McDonald-McGinn et al., 2015). In our study, all fetuses with 22q11.2DS presented with abnormal ultrasound findings. In contrast, only one of the seven fetuses (14.3%) with proximal 22q11.2 duplications was detected with structure defects, demonstrating the milder and highly variable phenotypes of proximal 22q11.2 duplications. The milder clinical phenotypes may contribute to the less number of duplication cases reported in literature compared with 22q11.2DS, although they are complementary to each other and predicted to occur at the same frequency (Portnoi, 2009). Recently, a case-cohort study in Danish population found that the prevalence of 22q11.2 microduplications was 1 in 1,606, about twice of 22q11.2 microdeletions, demonstrating distinct selective pressures on these rearrangements (Olsen et al., 2018). Of note, increased nuchal translucency was detected in three fetuses with proximal 22q11.2 duplications, which was in accordance with the study conducted by Celine and coworkers (about 37% fetuses presented with increased nuchal translucency) (Dupont et al., 2015). Previous studies had demonstrated that more than 90% of 22q11.2DS occurred *de novo*, and this was confirmed in our study (McDonald-McGinn et al., 2001). Our study also found 57.1% of proximal 22q11.2 microduplications were *de novo*, which was different from the previous reports (Van Campenhout et al., 2012). One reason for the inconsistency may be the small sample size in our study.

Compared with the well-defined 22q11.2DS, the reports of fetuses with central or distal 22q11.2 microdeletions/microduplications are limited. Previously, the central 22q11.2 deletions were recognized as “atypical/nested deletions” of 22q11.2DS (Rump et al., 2014; Verhagen et al., 2012; Garcia-Minaur et al., 2002). However, the *TBX1* gene, which was considered to be the major candidate gene for the main features of 22q11.2DS, was not included in the central 22q11.2 region, and recent studies proposed that the central 22q11.2 deletions were distinct form the 22q11.2DS (Burnside, 2015; Rump et al., 2014). Compared with patients with 22q11.2DS, patients with central 22q11.2 deletions had a lower prevalence of congenital heart defects while nearly equal prevalences of renal and urogenital anomalies, developmental delays, cognitive impairments, and behavioral problems (Rump et al., 2014; Verhagen et al., 2012). The *CRKL* (*602007) gene was thought to be the candidate gene in the pathogenesis of the 22q11.2 central deletion (Lopez-Rivera et al., 2017; Haller et al., 2017; Breckpot et al., 2012). Six 22q11.2 central deletions were detected in our study, indicating that central 22q11.2 deletions were common recurrent CNVs (Burnside, 2015). However, the prevalence of 22q11.2 central deletions in the general population has not been reported. To date, very few cases with central 22q11.2 duplications have been reported (Pebrel-Richard et al., 2012; Fernandez et al., 2009). The phenotypes of individuals with central 22q11.2 duplications were variable, ranging from clinically normal to severe developmental delay with profound intellectual disability. One recent study supposed the gene *PI4K* as a candidate gene responsible for the neurodevelopmental phenotypes in individuals with central 22q11.2 duplications (Woodward et al., 2019). Compared with the 22q11.2DS, most central deletions/duplications were familial in the reported cases, and our study was consistent with this (Rump et al., 2014; Clements et al., 2017). However, as the reported cases are limited, the proportion of *de novo* occurrence of this CNV needs to be studied further.

The four distal LCRs, LCR22E-H, were smaller than LCR22A-D, and the distal 22q11.2 deletions and duplications resulted from NAHR of them were less common compared with the 22q11.2DS (Coppinger et al., 2009). It was confirmed in our study that no fetus with distal 22q11.2 deletions and only two fetuses with 22q11.2 distal duplications were detected. The distal 22q11.2 microduplications or microdeletions were enriched in clinical population (Coe et al., 2014), and the phenotypes of individuals with distal 22q11.2 microdeletions or microduplications were variable with incomplete penetrance (Wincent et al., 2010; Tan et al., 2011). Mikhail et al. suggested the recurrent distal 22q11.2 microdeletions do not represent a single clinical entity and proposed to categorize them into three types with unique clinical features and risks according to their genomic position (Mikhail et al., 2014). Most of cases reported as distal 22q11.2 CNVs were in the region D–E/F and can be classified into type I microdeletions or microduplications (Mikhail et al., 2014). In contrast, few cases with type II/III microdeletions or microduplications have been reported, and the pathogenicity of distal type II/III 22q11.2 microdeletions or microduplications is unclear, calling for more case reports. In addition, the causal genes responsible for the phenotypes of the 22q11.2 distal microdeletions and microduplications are still unknown.

In our hospital, counseling on the 22q11.2 microdeletions or microduplications was provided by a geneticist in prenatal diagnosis center. We observed that 66.7% of the parents decided to terminate the pregnancy. Those with structure defects in prenatal ultrasound or occurred *de novo* often led to TOP, whereas those with normal ultrasound and inherited from healthy parent were likely to continue the pregnancy and led to normal birth. However, as 22q11.2 deletions/duplications were associated with many neuropsychiatric disorders including developmental delay, long-term monitoring and follow-up of these carriers were necessary.

In conclusion, our results exhibited the extreme variability of the 22q11.2 recurrent microdeletions and microduplications. Compared with the fetuses with 22q11.2 microduplications, fetuses with 22q11.2 microdeletions were more likely to present with structure defects in the ultrasound. Both the prenatal ultrasound findings and the inheritance of the CNVs affected the parent’s decision of pregnancy. Our study emphasized that proximal, central, and distal 22q11.2 deletions or duplications were different from each other, although some common features were shared among them. More studies are warranted to demonstrate the underlying mechanisms of different clinical features of these recurrent CNVs, thereby to provide more information for genetic counseling of 22q11.2 microdeletions and microduplications in prenatal diagnosis.

## Data Availability

The CNV data for this study can be found in the ClinVar database (https://www.ncbi.nlm.nih.gov/clinvar/, RCV000788056-RCV000788073).

## Ethics Statement

This study was carried out in accordance with the Declaration of Helsinki. The protocol was approved by the Ethics Committee of the IPMCH, and written informed consent was obtained from the parents.

## Author Contributions

SL drafted and revised the manuscript, with further contributions from XC. SL, XH, SC and YS performed the experiment. SL, XH, and XC carried out data analysis. YW and JN collected clinical sample and interpretation of clinical data. All authors were involved in the design and conception of the study, in addition to reading and approving the final manuscript.

## Funding

This work was supported by the National Natural Science Foundation of China (81871136, 81771638, 81501231), the Shanghai Municipal Commission of Science and Technology Program (16411963300), the Shanghai Municipal Health and Family Planning Committee (20164Y0212), the Interdisciplinary Program of Shanghai Jiao Tong University (YG2016MS40), the National Key Research and Development Program of China (2016YFC0905103), the International Peace Maternity and Child Health Hospital Clinical Research Project (GFY5817, GFY5818), and the Shanghai Municipal Key Clinical Specialty.

## Conflict of Interest Statement

The authors declare that the research was conducted in the absence of any commercial or financial relationships that could be construed as a potential conflict of interest.
