# Supplementary material for: Prenatal Diagnosis of Microdeletions or Microduplications in the Proximal, Central, and Distal Regions of Chromosome 22q11.2: Ultrasound Findings and Pregnancy Outcome
Source: Front Genet. 2019 Aug 30;10:813. doi: 10.3389/fgene.2019.00813 (PMC6728414; doi:10.3389/fgene.2019.00813)
Supplement: Supplementary file 1 [file Table_1.docx]

**Table S1**. The qPCR primers used in this study

| Region | Gene | Primer | | Length |
| --- | --- | --- | --- | --- |
| Proximal | *TBX1* | F | CGGGCAAGATAAAGAGCGGC | 101 |
|  |  | R | TGGGTAGCAACTTCCACGCA |  |
| Central | *CRKL* | F | GAAGTGAACGGGCGCAAAGG | 106 |
|  |  | R | GCAGCAGGAAACAGGGCAAC |  |
| Housekeeping gene | *HBB* | F | ACACAACTGTGTTCACTAGC | 110 |
|  |  | R | CAACTTCATCCACGTTCACC |  |

**Table S2**. The detail of cases included in this study

| ID | Referral indications | Maternal age (years) | Gestational age (weeks) | Specimen | CMA result | Length (Kb） | Inheritance | Karyotype result | Outcome |
| --- | --- | --- | --- | --- | --- | --- | --- | --- | --- |
| **22q 11.2 microdeletion** | | | | | | | | | |
| ***22q11.2 recurrent (DGS/VCFS) region (proximal, A-D) (includes TBX1)*** | | | | | | | | |  |
| 1 | Abnormal US findings: ventricle septal defect, obstruction of digestive tract, hydramnios | 26 | 25 | FB | arr[hg19] 22q11.21(18,922,151-21,449,911)x1 | 2528 | *de novo* | 46,XY | TOP |
| 2 | Abnormal US findings: endocardial cushion defect, overriding aorta, pulmonic stenosis | 20 | 24 | FB | arr[hg19] 22q11.21(18,916,842-21,798,907)x1 | 2882 | *de novo* | 46,XY | TOP |
| 3 | Abnormal US findings: right renal agenesis | 29 | 22 | AF | arr[hg19] 22q11.21(18,631,364-21,800,471)x1 | 3169 | *de novo* | 46,XY | TOP |
| 4 | Abnormal US findings: ventricle septal defect, pulmonary atresia | 28 | 22 | AF | arr[hg19] 22q11.21(18,648,855-21,800,471)x1 | 3152 | *de novo* | 46,XX | TOP |
| 5 | Abnormal US findings: right aortic arch, left ductus, aberrant left subclavian artery | 32 | 22 | AF | arr[hg19] 22q11.21(19,024,793-21,800,471)x1 | 2776 | *de novo* ^a^ | 46,XX | TOP |
| 6 | Abnormal US findings: Ventricular septal defect, truncus arteriosus persistens, persistent left superior vena cava | 25 | 24 | AF | arr[hg19] 22q11.21(18,631,364-21,800,471)x1 | 3169 | *de novo* ^a^ | 46,XX | TOP |
| 7 | Abnormal US findings: right renal agenesis, ventricular septal defect, overriding aorta | 34 | 26 | AF | arr[hg19] 22q11.21(18,636,749-21,800,471)x1 | 3164 | Maternal | 46,XX | TOP |
| 8 | Abnormal US findings: ventricular septal defect | 27 | 22 | AF | arr[hg19] 22q11.21(18,648,855-21,800,471)x1 | 3152 | *de novo* | 46,XX | TOP |
| 9 | Abnormal US findings: Tetralogy of fallot, right aortic arch, persistent left superior vena cava | 22 | 24 | AF | arr[hg19] 22q11.21(18,631,364-21,800,471)x1 | 3169 | *de novo* | 46,XY | TOP |
| 10 | Abnormal US findings: ventricular septal defect, persistent left superior vena cava | 36 | 25 | FB | arr[hg19] 22q11.21(18,919,477-21,800,471)x1 | 2881 | Paternal ^a^ | 46,XX | TOP |
| ***22q11.2 recurrent region (central, B/C-D) (includes CRKL)*** | | | | | | | | | |
| 11 | previous history of a fetus with structural anomaly | 33 | 20 | AF | arr[hg19] 22q11.21(20,716,876-21,800,471)x1 | 1084 | Maternal | 46,XY | Live birth with excrescence |
| 12 | Abnormal US findings: Dandy-Walker malformation, strephenopodia | 20 | 28 | FB | arr[hg19] 22q11.21(20,716,876-21,800,471)x1 | 1084 | Unknown | 46,XY | TOP |
| 13 | Abnormal US findings: ventricular septal defect，right aortic arch | 28 | 22 | AF | arr[hg19] 22q11.21(20,921,342-21,459,713)x1 | 538 | Unknown | 46,XX | TOP |
| 14 | Abnormal non-invasive prenatal testing | 27 | 18 | AF | arr[hg19] 22q11.21(20,716,876-21,800,471)x1 | 1084 | maternal ^a^ | 46,XY | Live birth |
| 15 | Abnormal US findings: ventricular septal defect | 40 | 26 | FB | arr[hg19] 22q11.21(21,033,586-21,800,471)x1 | 767 | Maternal | 46,XX | TOP |
| 16 | Abnormal US findings: double choroid plexus cysts, cerebellar vermis agenesis | 36 | 18 | AF | arr[hg19] 22q11.21(21,059,669-21,800,471)x1 | 741 | Unknown | 46,XY | TOP |
| **22q11.2 microduplication** | | | | | | | | | |
| ***22q11.2 recurrent (DGS/VCFS) region (proximal, A-B/D) (includes TBX1)*** | | | | | | | | | |
| 17 | Increased nuchal translucency (≥3.0 mm) | 28 | 18 | AF | arr[hg19] 22q11.21(18,912,403-21,801,520)x3 | 2889 | *de novo* | 46,XY | Live birth |
| 18 | Increased nuchal translucency (≥3.0 mm) | 30 | 12 | CVS | arr[hg19] 22q11.21(18,648,855-21,461,017)x3 | 2812 | *de novo* ^a^ | 46,XY | TOP |
| 19 | Increased nuchal translucency (≥3.0 mm) | 33 | 13 | AF | arr[hg19] 22q11.21(18,648,855-21,461,017)x3 | 2812 | *de novo* ^a^ | 46,XY | TOP |
| 20 | Advanced maternal age | 36 | 18 | AF | arr[hg19] 22q11.21(18,919,477-21,464,764)X3 | 2545 | Unknown | 46,XX | TOP |
| 21 | Advanced maternal age | 35 | 18 | AF | arr[hg19] 22q11.21(18,648,855-21,927,646)x3 | 3279 | paternal ^a^ | 46,XY | Live birth |
| 22 | Advanced maternal age | 39 | 18 | AF | arr[hg19] 22q11.21(18,648,855-21,454,872)x3 | 2806 | unknown | 46,XX | Live birth |
| 23 | Abnormal US findings: ventricular septal defect | 29 | 21 | AF | arr[hg19] 22q11.21(18,937,380-21,459,713)X3 | 2522 | *de novo* | 46,XX | TOP |
| ***22q11.2 recurrent region (central, B-D) (includes CRKL)*** | | | | | |  |  |  |  |
| 24 | Advanced maternal age | 40 | 21 | AF | arr[hg19] 22q11.21(20,992,308-21,464,764)x3 | 472 | paternal | 46,XY | Live birth |
| 25 | Advanced maternal age | 35 | 19 | AF | arr[hg19] 22q11.21(20,729,388-21,454,872)x3 | 725 | maternal ^a^ | 46,XY | Live birth with acleistocardia and tricuspid valve regurgitation |
| ***22q11.2 recurrent region (distal)*** | | | | | | | | | |
| 26 | Positive second-trimester  screening | 33 | 19 | AF | arr[hg19] 22q11.23(23,652,517-25,002,659)x3 | 1350 | maternal ^a^ | 46,XY | Live birth |
| 27 | Second-trimester sonographic marker：echogenic bowel | 30 | 23 | AF | arr[hg19] 22q11.22q11.23(22,997,928-23,654,007)x3 | 656 | unknown | 46,XX | Live birth |

^a^ The inheritance was known before the decision making.

US, ultrasound; CMA, chromosomal microarray analysis; FB, fetal blood; AF, amniotic fluid; CVS, chorionic villus sampling; TOP, termination of pregnancy.
